# Reference‐dependent age weighting of quality‐adjusted life years

**DOI:** 10.1002/hec.4593

**Published:** 2022-09-04

**Authors:** Arthur E. Attema, Werner B. F. Brouwer, Jose Luis Pinto‐Prades

**Affiliations:** ^1^ Erasmus School of Health Policy & Management (ESHPM) Erasmus University Rotterdam the Netherlands; ^2^ Faculty of Economics Universidad de Navarra Pamplona Spain

**Keywords:** age weighting, equity weighting, quality‐adjusted life years, reference‐dependence

## Abstract

People do not only care about maximizing health gains but also about their distribution. For example, they give more weight to younger patients than older patients. This pilot study aims to investigate if age weighting is reinforced by loss aversion if young people are falling behind one's perceived ‘normal’ quality of life (QoL), while older people do not. We apply a person trade‐off method in a large representative sample (*n* = 990) to estimate age weighting factors. We also measure QoL levels that individuals regard as ‘normal’ for different ages, serving as reference points. We observe a considerable amount of age weighting, with 20‐year‐old patients on average receiving 1.7 times as much weight as 80‐year‐old patients. Perceived ‘normal’ QoL rapidly decreases with age of a patient. Older people are more optimistic about what constitutes ‘normal QoL’ than younger people, but they express a faster decline in normal QoL due to aging. Respondents who view all improvements to be gain enlarging show the least age weighting, but loss aversion cannot explain the results. Still, one's age‐related reference level is an important predictor of age weights. Given the explorative nature of this study, further studies are called for to generate more robust evidence.

## 1 INTRODUCTION

The rising healthcare costs that result, amongst other things, from an aging society and improved, more expensive, medical technology have been a cause of concern for many years (Fuchs, 2011). Recently, the COVID‐19 virus has led to an even stronger pressure on most countries' healthcare capacities. These developments have highlighted the need for health economic evaluation to guide the funding of new drugs and treatments. The growing demand for healthcare also fuels concerns that not all patients can always rely on receiving necessary treatments and highlights the difficult trade‐offs that need to be made in the healthcare sector. The fact that intensive care facilities might reach their maximum capacity during a pandemic, requiring rules to ration intensive care, may illustrate this point (Rosenbaum, 2020; Supady et al., 2021). While such examples may be relatively extreme, choices made on a day‐to‐day basis in allocating scarce healthcare resources, require normative rules. Next to well accepted considerations such as the effectiveness of treatments offered, other considerations can include their efficiency (or cost‐effectiveness) as well as equity considerations.

In that context, age of patients has also been addressed in the literature as a potentially relevant element to consider in allocating scarce healthcare resources (Dolan et al., 2005; Tsuchiya, 1999). In particular, it has frequently been suggested that one could or even should give priority to young patients over old (Persad et al., 2009; Williams, 1997). Such a priority could be based on considerations of effectiveness, because a treatment may be more effective in younger people, or because young people have a higher life expectancy and, hence, may gain more quality‐adjusted life years (QALYs) from a cure or life‐saving treatment than older people (Gu et al., 2015). Such considerations would, ceteris paribus, also lead to a more favorable cost‐effectiveness in younger versus older patients. Considering age in healthcare priority setting may moreover be based on equity concerns, for instance because the young have not yet had their “fair share” of health yet or would fall behind what would be considered a “normal” or average health expectation (Williams, 1997). In those circumstances, age weighting could be applied to express such concerns, where 1 QALY gained in a young person would receive more weight than 1 QALY gained in an older person in decision making.

It is important to emphasize that it has been recognized that standard cost‐utility analyses, that use unweighted QALYs as a proxy for utilities, do not seem to fully reflect societal values, and thus may misrepresent the goals and informational needs of policy makers (Coast, 2004; Dolan et al., 2005; Nord et al., 1999). Indeed, empirical evidence suggests that the goal of maximizing the number of QALYs, regardless of their distribution, should not be the sole objective of health care policies. Many individuals and policy makers also have preferences for a particular distribution of those QALYs, and thus are willing to make trade‐offs between the goals of equity and efficiency when making health care decisions (Tsuchiya & Dolan, 2005; van de Wetering et al., 2013). Age appears to be an important element in that context, even when using equity principles such as proportional or absolute shortfall. First, depending on the impact of a disease on quality and length of life, older patients may be more or less likely to be classified as having a disease of high severity than young patients, because the former have a lower remaining life expectancy (Reckers‐Droog et al., 2021). Second, young patients may get more weight in decision making than old patients even when severity is the same. For example, Reckers‐Droog et al. (2019) showed that the general public gives priority to younger patients when disease severity is equal, while Reckers‐Droog et al. (2021) found that people are willing to pay more for health gains in younger than older end‐of‐life patients when controlling for disease severity. However, how age weighting relates to age‐related health perceptions can still be investigated further.

In this study, we consider the role of age‐specific reference points for perceived normal health‐related quality of life (QoL), since health tends to deteriorate over time during one's life, causing people to have particular expectations regarding their health state at different ages (Brouwer et al., 2005; Stolk et al., 2002; Tsuchiya, 2000; Williams, 1997). Indeed, previous empirical research found that people form reference points of health levels that are considered as “acceptable” for specific age groups (Brouwer et al., 2005; Wouters et al., 2015; Zrubka et al., 2019). For example, an 80‐year‐old person is not expected to have the same health state as a 20‐year‐old person. Consequently, the reference point for the 80‐year‐old person's health may be considerably lower than the reference health state of the 20‐year‐old person. A robust finding in these studies was that the health state that people perceived as “acceptable” or “normal” decreased rapidly for older ages (Attema et al., 2015; Brouwer & van Exel, 2005; Péntek, Brodszky, et al., 2014; Rappange et al., 2016; Wouters et al., 2015; Zrubka et al., 2019). Age weighting or observed age‐related distributional preferences may then (also) relate to whether or not people fall below what is considered normal QoL at a given age.

In this study we further investigate this question, with an emphasis on the role of reference points in terms of what is considered normal QoL over the lifecycle. We extend the aforementioned studies in several directions. First, we elicit age weights given to health improvements of specific age groups and test if these weights are related to perceived normal QoL levels at different ages. Second, we elicit age weights for two different initial health levels, in order to test if these age weights are robust to the severity of the health states. We do the same for the duration of the health improvement, type of health problem (mental or physical), and description of the condition (EuroQol's EQ‐5D classification system or the Health Utilities Index [HUI3]). In what follows, Section 2 introduces notation and describes the theoretical background, Section 3 describes the experimental design, Section 4 presents the results, and Section 5 provides a discussion and concludes.

## 2 METHOD

We consider a population of two groups of individuals and let (*q*_y_, *q*_o_) denote a profile that indicates the QoL *q*, as described by some classification system (see Section 3), of each individual of groups *y* and o, respectively, where *y* denotes young, and o denotes old. Each group consists of the same number of individuals at a given age. The only difference between the groups is the age of their members.

We assume that the health‐related social welfare function represents the social value of the health profile (*q*_y_, *q*_o_) by:

(1) $$W=w_{y}U\left(q_{y}\right)+\left(1-w_{y}\right)U\left(q_{o}\right)$$

where the *w*_i_ are age weights, with *w*_y_ + *w*_o_ = 1, and $U\left(q_{i}\right)$ is a policy maker's utility function over QALYs, as in, among others, Bleichrodt (1997), Dolan (1998), and Bleichrodt et al. (2005).

Furthermore, we capture reference‐dependent age weighting by the utility function:

(2) $$U\left(q_{i},t\right)=\left\{\begin{array}{ll} u\left(q_{i}\right)\times t & \text{if}\;q\geq q^{*}\left(a\right)\\ u\left(q^{*}\left(a\right)\right)\times t+\lambda \left[u\left(q_{i}\right)-u\left(q^{*}\left(a\right)\right)\right]\times t & \text{if}\;q<q^{*}\left(a\right) \end{array}\right.$$

That is, U(*q*_i_,*t*), with *t* the duration of *q*_i_ in years, is reference‐dependent, with separate functions for gains and losses in QoL. The utility function u(*q*_i_) assigns a numerical value to health state *q*_i_, and is the same for gains and losses, albeit that if a group's *q* is falling below their age (a)‐dependent reference level (RL) q*(a), the gap between *u*(*q*_i_) and *u*(q*(a)) is penalized by a factor *λ* > 1 (Shalev, 2002). This reflects the concept of loss aversion, as part of prospect theory, where losses get more weight than commensurate gains (Tversky & Kahneman, 1991). Prospect theory was originally developed to describe individual choice, but its insights, in particular the concept of reference‐dependence, can also be applied to social choice, as shown by Dolan and Robinson (2001), Bleichrodt et al. (2004) and Attema et al. (2015) for health outcomes. Numerous studies have found evidence of loss aversion, not only for money (Starmer, 2000; Wakker, 2010), but also for health, both from the individual perspective (Attema et al., 2013, 2018; Lipman et al., 2019; Rouyard et al., 2018) and from the societal perspective (Attema et al., 2015).

In our study, one group consists of 100 patients of the same age, while the other group consists of 100 patients of a higher age (but again all the members within this group have the same age). With this weighting function, a general preference for the younger group (*y*) over the older group (*o*) can be modeled by, for instance *w*_y_ = 0.6 and *w*_*o*_ = 0.4. This means the young get 0.6/0.4 = 1.5 as much weight as the old.

If some group has a health state which is worse than what is considered normal for this group's age, it would be seen as being in a loss situation. Therefore, any health improvement in this group could be given more weight than the same health improvement in some other group with the same health status, because they are older and this health status is considered better than their normal QoL. Note that although both age weighting caused by a separate equity function, and age weighting caused by the QALY model (e.g., because young people have a higher life expectancy), may result in the young person receiving priority over the old person, the underlying reasons are fundamentally different. The first reflects a “deserving” preference for giving QALYs to younger instead of older people, for example, motivated by the fair innings argument, whereas the second reflects a preference for giving a QALY to the group or person in a loss situation instead of giving it to the group or person in a gain situation. Hence, the latter is not necessarily age‐driven, although it might very well have similar consequences in case of age‐related reference points.

Let us clarify this distinction with an example: imagine the perceived normal QoL status for the young group is full health (i.e., q*(y) = full health, with *u*(q*(y)) = 1), whereas the perceived normal QoL status for the old group is q*(o), which when lasting for 1 year has utility *u*(q*(*o*)) = 0.5. The weight given to a QALY gained in each group depends on the current health of the group members. If *u*(*q*_i_) = 0.7 in both groups, then a utility increase from 0.7 toward 0.8 will be considered a gain for the old group, but seen as a loss reduction for the young group. Due to loss aversion, the improvement will receive more weight for the young group. For example, if *λ* = 2, Equation (2) gives Δ*u*(*q*_y_) = 2 × (0.8 – 0.7) = 0.2, versus Δ*u*(*q*_*o*_) = 0.8 – 0.7 = 0.1. If *w*_*y*_ = *w*_*o*_ = 0.5, the increase in social welfare due to the health improvement for the young (old) group would be 0.1 (0.05).

## 3 EXPERIMENT

### 3.1 Design and participants

We ran an experiment consisting of six versions. Ethical approval for this study was provided by the Research Ethics Review Committee of Erasmus University. The results reported in this paper are part of a larger study (see Attema et al. (2022) for the other part of the study). Questions on socio‐demographics and reference points were asked to all respondents. We implemented a modified version of the person trade‐off (PTO) task (Nord, 1992; Reckers‐Droog et al., 2019), where respondents had to consider a fixed budget that could be allocated to treat one of two groups of people. The groups were the same in all aspects, except for the age of their members. The patients in both groups suffered from an illness which would improve after 6 months or 5 years (depending on the version) with standard treatment. However, the budget would allow to give a new treatment to only one of these groups. With this treatment, the health improvement would be accelerated by 3 months (6‐month scenario) or by 4 years and 9 months (5‐year scenario). In the first choice, both groups consisted of 100 patients. If the respondent chose to treat the young (old) group, the number of patients in the younger (older) group was decreased.

After four dichotomous iterative choices, we showed a scroll bar to the respondents, which was censored to the range as implied by the respondents' choices, allowing them to pick an indifference value within this range. The initial health level was the same for the two groups in each question but varied between questions.

The RP was elicited by presenting the description of each level of the health dimension and asking which level the respondent deemed to be normal for a 20‐year‐old and an 80‐year‐old. Appendix A presents the instructions and examples of each type of questions asked in the experiment.

### 3.2 Respondents and procedure

The questionnaire was completed by a representative sample of the Dutch general public between 18 and 86 years in terms of age, gender, education and geographic spread (*n* = 1000). The experiment was programmed in Qualtrics and administered by a professional Internet sampling company (Nexo). This company has a large representative database of respondents. The respondents were rewarded with points that they could accumulate to receive a small gift. Respondents were randomly allocated to one of the six versions (see Table 1), with the restriction that each version was completed by the same number of men and women, and an equal division of respondents in the age groups 18–44, 45–60 and > 60 years, which contain approximately the same number of people in the Netherlands (Statistics Netherlands).

**TABLE 1:** Overview of implied type

	Loss reduction for 80‐year‐old	Gain enlargement for 80‐year‐old
Loss reduction for 20‐year‐old	Type 2	Type 1
Gain enlargement for 20‐year‐old	Type 4 (not studied)	Type 3

We started the experiment with some questions regarding demographic characteristics. We gathered information about age, gender, education, and rating of own health (according to a visual analog scale). Elaborate instructions and several practice questions preceded the main experiment. In order to guarantee sufficient understanding of the task among respondents, they were given a final check at the end of the practice questions where one option dominated the other. If they chose the dominated option here, they received feedback explaining that they chose a dominated option and were asked if they wished to reconsider their answer to this final check. One of the questions was repeated at the end of the questionnaire to test reliability of the answers.

In each choice in the PTO task, respondents were asked to consider N_y_ patients in group 1 to N_o_ patients in group 2. The patients in one of these groups could get an acceleration of a medical treatment that would expedite an improvement of their health state from *q*_i_ to ${q_{i}}^{\prime }$. For example, consider the question where *y* = 20, *o* = 80, and the treatment was accelerated by 3 months (i.e., 0.25 years). Given the values of N_20_ and N_80_ for which the respondent was indifferent between treating the two groups, the following equation results from Equation (2):

(3) $$N_{20}\times \left(U\left(q_{i}^{\prime },0.25\right)-U\left(q_{i},0.25\right)\right)\times w_{20}=N_{80}\times \left(U\left(q_{i}^{\prime },0.25\right)-U\left(q_{i},0.25\right)\right)\times \left(1-w_{20}\right)$$

where $\left(U\left(q_{i}^{\prime },0.25\right)-U\left(q_{i},0.25\right)\right)$ is the amount of the social utility gain caused by a QoL improvement from *q*_I_ to *q*_i_′ during 0.25 years. Either side of the equation represents the equity‐weighted incremental QALY gain to society from the treatment acceleration. For the longer duration, 0.25 can be replaced with 4.75. Because this factor cancels out of Equation (3), duration does not matter in this design when our model holds, and we suppress it in the following derivations. If we find systematic differences between these two durations, this suggests the model and, hence, Equation (3) is not valid. For instance, it has been found that a proportion of respondents tends to disregard relevant information in ageism elicitation studies, which might cause framing effects not predicted by our model (Tsuchiya et al., 2003). Now, we introduce four possible types of respondents, based on their views on what is normal QoL.

*Implied Type 1: Gain enlargements for the old, loss reductions for the young*

The first scenario is where the reference QoL level is higher for 20‐year‐old individuals than 80‐year‐old individuals, such that the former are in the loss domain, whilst the latter are in the gain domain. The health improvement will then be evaluated as a loss reduction in the young group, and as a gain enlargement in the old group. Hence, we obtain the following equation representing indifference:

(4) $$N_{20}\times \lambda \left(u\left({q_{i}}^{\prime }\right)-u\left(q_{i}\right)\right)\times w_{20}=N_{80}\times \left(u\left({q_{i}}^{\prime }\right)-u\left(q_{i}\right)\right)\times \left(1-w_{20}\right)$$

The utility gain of the health improvement cancels out in this scenario, so that we can easily compute *N*_20_:

(5) $$N_{20}=N_{80}\left(\frac{1-w_{20}}{\lambda w_{20}}\right)$$

An intermediate possibility is where the young group starts off in the loss domain, but ends up in the gain domain after the treatment, while the old group is in the gain domain from the beginning. This results in a more complicated indifference evaluation, since part of the improvement of the young patients is a loss reduction, and part is a gain enlargement:

(6) $$N_{20}\times \left(\lambda \left(u\left(q^{*}\right)-u\left(q_{i}\right)\right)+\left(u\left({q_{i}}^{\prime }\right)-u\left(q^{*}\right)\right)\right)\times w_{20}=N_{80}\times \left(u\left({q_{i}}^{\prime }\right)-u\left(q_{i}\right)\right)\times \left(1-w_{20}\right)$$

Solving for *N*_20_ yields:

(7) $$N_{20}=N_{80}\left(\frac{1-w_{20}}{w_{20}}\right)\left(\frac{u\left({q_{i}}^{\prime }\right)-u\left(q_{i}\right)}{\lambda \left(u\left(q^{*}\right)-u\left(q_{i}\right)\right)+u\left({q_{i}}^{\prime }\right)-u\left(q^{*}\right)}\right)$$

This option is qualitatively similar to the previous one, albeit that the impact of loss aversion is smaller, as it influences only part of the health improvement for the young group. Therefore, in what follows, we consider this special case separately.

*Implied Type 2: All loss reductions*

The second type of respondent has a high reference QoL level for both age groups, resulting in both health improvements being evaluated in the loss domain. Indifference is then evaluated by the following equation:

(8) $$N_{20}\times \lambda \left(u\left({q_{i}}^{\prime }\right)-u\left(q_{i}\right)\right)\times w_{20}=N_{80}\times \lambda \left(u\left({q_{i}}^{\prime }\right)-u\left(q_{i}\right)\right)\times \left(1-w_{20}\right)$$

Loss aversion cancels out in this equation, so we obtain the following expression for N_20_:

(9) $$N_{20}=N_{80}\left(\frac{1-w_{20}}{w_{20}}\right)$$

Hence, any difference between N_20_ and N_80_ is determined by the amount of age weighting (with N_20_ and N_80_ the same if *w*_20_ = 0.5). Comparing to Type 1, we see that in case of loss aversion, *λ* > 1 and, hence, N_20_ is smaller for Type 1 than for Type 2 for the same values of N_80_ and *w*_20_. In other words, fewer young patients have to be treated for Type 1 respondents in order to bring the same social welfare gain as treating a given number of old patients.

*Implied Type 3: All gain enlargements*

The situation where both groups are in the gain domain gives the same prediction as for Type 2, since the loss domain and, hence, *λ* are not relevant in this case.

*Implied Type 4: Gain enlargements for the young, loss reductions for the old*

The final type is where the health improvement is regarded as a gain enlargement for 20‐year‐olds and a loss reduction for 80‐year‐olds. This yields the opposite prediction of Type 1, with young patients getting treatment for *λ* > 1:

(10) $$N_{20}=N_{80}\lambda \left(\frac{1-w_{20}}{w_{20}}\right)$$

However, this type is not intuitive because it implies the normal QoL of an 80‐year‐old would be seen as better than that of a 20‐year‐old. Indeed, as shown in the Results Section, such a perception was rare, and we do not pursue it further. The same holds for the intermediate case in this type, with partial loss reductions and partial gain enlargements for the old. We summarize the four respondent types in Table 1.^1^

### 3.3 Hypotheses

We predict that there is a stronger preference for the young in case of patients with mild health problems than in case of patients with severe health problems. The reason for this is related to reference‐dependence. Specifically, given that most people would consider it normal for young people to be in good health, whereas it would be regarded as more acceptable for older people to have some health issues (Brouwer et al., 2005; Wouters et al., 2015), mild health problems may be felt as losses for young people, but as normal or even gains for the elderly. If respondents are loss averse with respect to health levels that fall short of these “age‐specific reference points”, they will assign more weight to health improvements for the young than for the old (in accordance with Equation (5)). Our expectation is that for mild problems, it is more likely that older people are above the reference point of normal QoL, and younger people are below it. For severe problems, it is instead more likely that both the old and the young are below their perceived normal QoL. Hence, Type 1 would be more common for mild problems, and Type 2 would be the most common scenario for severe initial health states.

Second, we test if there is a difference in age preferences for health problems that last longer without the new treatment. Therefore, we include two variations in the duration of the health problems for the group not getting the best treatment: one where the problems last for another 6 months and one where they last for 5 years. According to Equation (3), duration should not matter. If instead it matters, this suggests the model is not valid, and additive separability is violated.

Third, we predict that age weighting is smaller for mental health problems than for physical problems. The reason is that physical health normally deteriorates with age, whereas for mental health, this relation with age is less straightforward. Consequently, it is possible that Type 2 (or 3) is more relevant for mental health problems, while Type 1 is more relevant for physical problems, resulting in more age weighting for the latter in case of loss aversion.

### 3.4 Stimuli: health state description

We described two health conditions in terms of problems on the dimensions mobility and anxiety/depression of EuroQol's EQ‐5D‐5L system (Herdman et al., 2011). For both anxiety and mobility, we used two severity levels: 3 (some problems) and 5 (extreme problems). For starting level 3, groups of patients could improve to level 1 (no problems), and for starting level 5, they could improve to level 3. Because level 1 (3) is strictly better than level 3 (5), we assume that U(level 1) > U (level 3) > U(level 5). In terms of Equation (3), if we have *q* = anxiety at level 3 and q’ = anxiety at level 1, then it follows that U(*q*’)>U(*q*). These utilities are usually assigned to EQ‐5D health states by using specific national tariffs estimated by means of time trade‐off tasks and modeling (e.g., Devlin et al., 2018; Versteegh et al., 2016), but for our purpose the only requirement is that U(*q*’) > U(*q*) and a precise estimate of U(*q*) is not needed.^2^

We also described two conditions in terms of attributes of the HUI3 (Horsman et al., 2003). The HUI3 consists of eight attributes each decomposed into six levels. Out of these eight attributes, we selected the attributes ambulation and hearing. Ambulation involves the ability to walk around the neighborhood, whilst hearing is described by different abilities to hear what is being said in conversations. A complete description of these two attributes of the HUI3 classification system is shown in Appendix A. For both attributes, we used improvements from level 2 to level 1, and from level 4 to level 2. The order of the starting levels was randomized within‐subjects.

For EQ‐5D‐mobility and HUI3‐hearing, we also implemented a variation with a longer duration of the health improvements. In order to estimate what health level the respondents perceived as normal at different ages, we asked what level of each of the dimensions used (i.e., EQ‐mobility, EQ‐anxiety, HUI‐ambulation and HUI‐hearing) they deemed normal at the ages of 20 and 80. As a result of the aforementioned variations, we could test for the effects of duration, severity, classification system (mobility in EQ‐5D vs. ambulation in HUI3) and mental versus physical health problems (EQ‐anxiety vs. EQ‐mobility) on age weighting, Table 2 gives an overview of the tasks included in each of the six versions of the experiment.

**TABLE 2:** Overview of the different versions

Task	Version A	Version B	Version C	Version D	Version E	Version F
Delay of health improvement	3 months or 6 months	3 months or 6 months	3 months or 6 months	3 months or 6 months	3 months or 5 years	3 months or 5 years
EQ‐5D—mobility	3→1				3→1	
	5→3				5→3	
EQ‐5D—anxiety			3→1			
			5→3			
HUI—ambulation				2→1		
				4→2		
HUI—hearing		2→1				2→1
		4→2				4→2

Abbreviation: HUI, Health Utilities Index.

### 3.5 Analysis

Given a lack of an estimate of loss aversion, we start from the assumption that *λ* = 1. In the analysis of age weighting, we use the estimated values of *w*_20_, computed by solving Equation (9) for *w*_20_:

(11) $$w_{20}=\frac{N_{80}}{N_{20}+N_{80}}$$

If we now find a higher value of *w*_20_ in the between‐subject comparisons for Type 1 than for Types 2 and 3, we attribute this to loss aversion, because according to our model loss aversion is predicted to affect Type 1 (Equation (5)), but not Types 2 and 3 (Equation (9)). That is, one could find a value of *λ* > 1 such that the adjusted values of *w*_20_ are the same for these scenarios. Furthermore, a value of *w*_20_ > 0.5 (<0.5) signals a preference for the young (old) group.

For the improvement from Level 3 to Level 1 in EQ‐5D, the improvements were in the gain region for those with an RL of 3–5 and in the loss region for those with RL = 1. For those with RL = 2, the gain was partly loss reducing and partly gain enlarging. As such, we analyze this task comparing those with RL = 1 to those with RL = 2–5 on the one hand, and those with RL = 1–2 to those with RL = 3–5 on the other hand. Because the former generated a very small number of respondents of Type 2, we focus on the latter. The results of the alternative classification are comparable and reported in Table B2 of Appendix B. Similarly, the increase from five to three is loss reducing for RL = 1–3, gain enlarging for RL = 5 and a combination of both for RL = 4. Hence, we compare those with RL = 1–3 to those with RL = 4–5 and do a second analysis comparing those with RL = 1–4 with RL = 5 (reported in Table B3 of Appendix B).

For HUI3, the improvement from two to one was loss reducing for respondents with RL = 1 and gain enlarging for those with RL = 2–6. Finally, the improvement from four to two is loss reducing for those with RL = 1–2, gain enlarging for those with RL = 4–6 and a combination for those with RL = 3. Hence, we compare respondents with RL = 1–3 to those with RL = 4–6 in the first analysis, and report summary statistics for the comparison of RL = 1–4 to RL = 5–6 in Table B3.

Apart from these between‐subject comparisons, we also compare the age weights for mild and severe initial health states within‐subjects. We make these comparisons for the different types. For the severe initial health state, as we see in the Results section, most respondents regarded the health improvements as Type 2, with both groups being in the loss domain. For the mild initial health states, respondents were more divided between the different types. Therefore, we compare the age weights for the mild initial health state with those for the severe initial health state separately for respondents of Type 1, 2 and 3. Corresponding to loss aversion, we expect a larger difference between the amounts of age weighting for Type 1 than for the other Types. We use either paired *t*‐tests (within‐subjects) or independent sample *t*‐tests (between‐subjects).

As it turns out that the number of respondents in each type is somewhat unbalanced, we also perform analyses grouping all versions together. This means we aggregate age weights for different health conditions, but it increases the statistical power for testing for reference effects.

## 4 RESULTS

The respondents were almost equally distributed between the six versions. Each version was completed by the same number of men and women, and for one third by people in the group 18–44 years, for one third by the age group 45–60 years old and for one third by people older than 60 years. Seven respondents indicated to be younger than 18 years and were excluded from the analysis. Three other respondents did not complete the PTO task and were excluded from the analysis as well. Hence, the final sample included 990 respondents. The Internet panel members were geographically spread through the country in accordance with population density and education levels were similar (Statistics Netherlands). Table 3 shows demographic characteristics of our sample. These characteristics were similar for the six versions.

**TABLE 3:** Demographic characteristics (full sample, *n* = 990)

Variable	Percentage	Mean	SD	Minimum	Maximum
Age of respondent		50.96	14.72	18	86
Gender (% male)	49.9				
Education:					
Lower	26.3				
Middle	39.6				
Higher	34.1				
Health status: VAS		74.12	16.98	0	100
Completion time (mins.)		24′ 10″	78′21″	2′21″	34h

Abbreviation: VAS, visual analog scale.

### 4.1 Data quality

The dominated option of the practice question was chosen 19.2% of the times. Out of those, 35.3% (*n* = 82) reversed their choice after receiving feedback. Hence, the feedback provision was not very successful in improving task comprehension. Further, 47 respondents performed the survey in less than 5 min, which is very fast given the number of tasks and suggests speeding through the survey.^3^ Respondents violating dominance even after receiving feedback and/or completing the survey in less than 5 min were excluded from the dataset in a robustness analysis (Web Appendix), which did not change the main results.^4^ The repeated question was answered the same by 79.9% of the respondents, resulting in a high test‐retest reliability.

### 4.2 Perceived normal QoL levels

The overview of perceived normal levels shows no clear distinction between the different health attributes (Figure 1 and Table 4). For all domains, level 1 is the modal level for 20‐year‐olds, whereas level 3 is the modal level for 80‐year‐olds. Still, in agreement with our expectation, respondents indicated that the distinction between physical and mental health states was significant, with mental health problems more often being considered to be normal at age 20 and mobility problems more often being considered normal at age 80 (*p*'s < 0.01). Furthermore, on the HUI3 scale, we found hearing problems to be considered normal more often than ambulation problems at age 80 (*p* < 0.02).

FIGURE 1Frequency distributions of perceived normal levels for different attributes of EQ‐5D and Health Utilities Index (HUI). Panel (a) Relative frequencies of perceived normal levels of mobility from the EQ‐5D‐5L system for 20‐year‐olds and 80‐year‐olds (measured in Version A and E). Panel (b) Relative frequencies of perceived normal levels of anxiety/depression from the EQ‐5D‐5L system for 20‐year‐olds and 80‐year‐olds (measured in Version C). Panel (c) Relative frequencies of perceived normal levels of ambulation from the HUI‐3 system for 20‐year‐olds and 80‐year‐olds (measured in Version D). Panel (d) Relative frequencies of perceived normal levels of hearing from the HUI‐3 system for 20‐year‐olds and 80‐year‐olds (measured in Version B and F)

**Figure:**
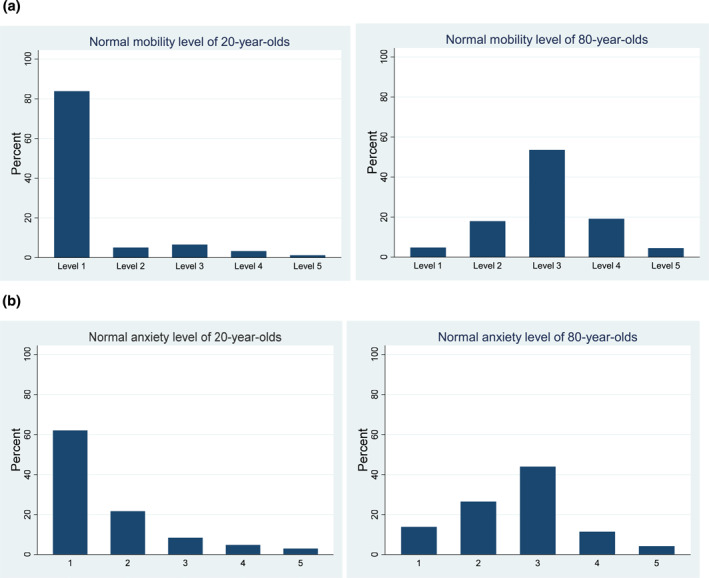

**Figure:**
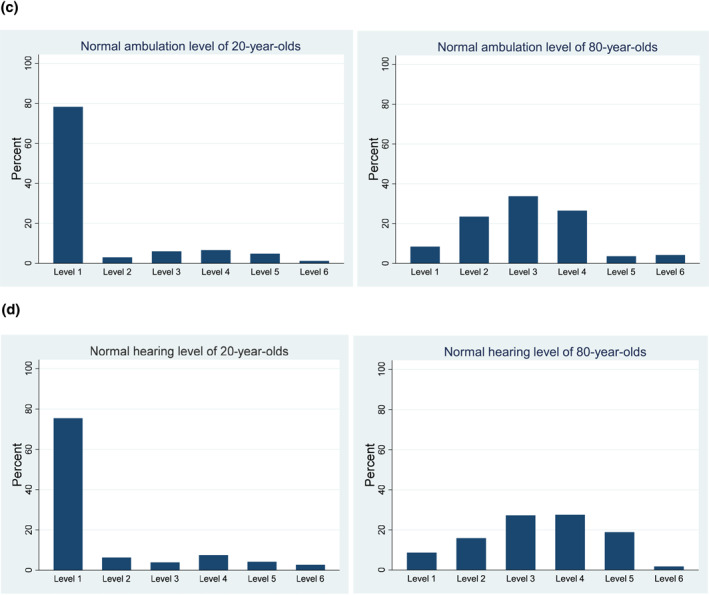

**TABLE 4:** Average perceived normal levels per attribute for 20‐ and 80‐year‐olds (standard deviations in parentheses)

Age of patients	Mobility (scale 1–5)	Anxiety/Depression (scale 1–5)	Hearing (scale 1–6)	Ambulation (scale 1–6)
20 years	1.33 (0.84)	1.63 (1.01)	1.67 (1.34)	1.60 (1.26)
80 years	3.01 (0.86)	2.67 (1.00)	3.37 (1.25)	3.06 (1.17)

The perceived normal QoL levels are strongly negatively correlated with age of respondents (Pearson correlation, *p* < 0.01). Hence, older respondents are more optimistic about what can be considered normal QoL. Interestingly, this holds both for indications of normal QoL for 20‐year‐olds and those for 80‐year‐olds. This suggests that older people are not only more optimistic about what is normal QoL at older ages, but instead about what is normal QoL in general. This could of course also be the consequence of (retrospectively applying) adaptation, or of stating a higher level of QoL at age 80 ‐ assuming a negative age gradient for perceived normal QoL necessarily results in a higher starting point at age 20. In that context, it is relevant to observe that while older respondents are more optimistic about normal QoL at ages 80 and 20, they do expect the largest deterioration in that period; the amount of deterioration of perceived normal QoL between 20‐year and 80‐year is significantly positively correlated with respondents' age (Pearson correlation, *p* < 0.01). This is possible because the difference in perceived normal QoL at age 20 is larger than at the age of 80.

### 4.3 Age weights

Table 5 presents the average values of *w*_20_ for all tasks (quartiles are reported in Table B1 in Appendix B). These numbers show preferences for treating young respondents in all questions. All weights are significantly higher than ½ (*p* = 0.024 for a hearing improvement from level 4 to level 2 and *p* < 0.01 for all other tasks). We find no difference between mental and physical health improvements (*p* > 0.70). For severity, we only observe a difference for the short improvement duration for hearing (Version B), with less weight given to the young for the severe initial level than the mild initial level (*p* < 0.01; all other *p*'s > 0.10). This age weighting found for hearing in Version B is also stronger than for the longer duration of Version F, again only for the mild initial health state (*p* < 0.01 vs. *p* = 0.70). No such effect of duration was found for mobility (A vs. E, *p* < 0.43). For the full dataset, with all conditions combined, we find a difference between the mild and the severe condition, which was significant only at the 10% level (mean *w*_20_ = 0.63 for mild and 0.62 for severe, *p* = 0.074). No significant correlation is found between the age of the respondent and age weight for the mild condition (Pearson correlation, *p* = 0.11), but a positive significant correlation is present for the severe condition (*p* = 0.027).

**TABLE 5:** Mean estimates of *w*_20_ (standard deviations in parentheses)

Delay of health improvement				
	3 months or 6 months		3 months or 5 years	
	3→1 (EQ5D)	5→3 (EQ5D)	3→1 (EQ5D)	5→3 (EQ5D)
Health improvement	2→1 (HUI3)	4→2 (HUI3)	2→1 (HUI3)	4→2 (HUI3)
Task				
EQ‐5D—mobility	0.61 (0.28)	0.61 (0.28)	0.61 (0.28)	0.64 (0.28)
EQ‐5D—anxiety	0.62 (0.27)	0.62 (0.25)		
HUI—ambulation	0.65 (0.25)	0.63 (0.26)		
HUI—hearing	0.64 (0.24)	0.55 (0.28)	0.65 (0.26)	0.65 (0.27)

Abbreviation: HUI, Health Utilities Index.

**FIGURE 2:**
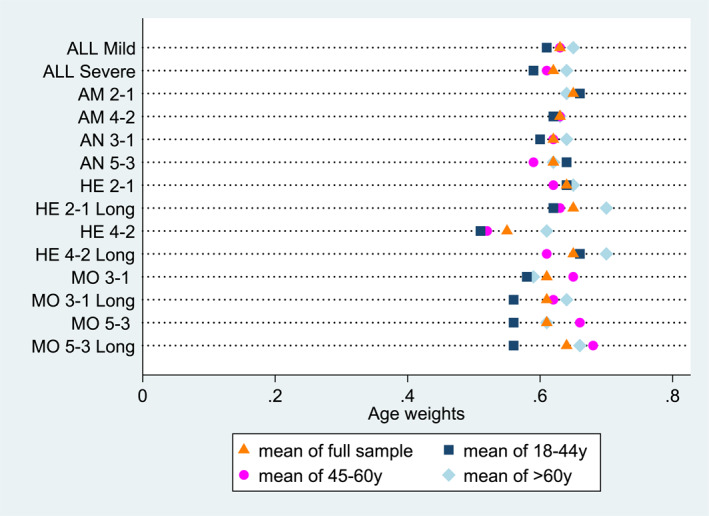
Mean age weights by respondents' age group. AM, Ambulation; AN, Anxiety; HE, Hearing; MO, Mobility; ALL Mild, all weights combined for the mild initial health state; ALL Severe, all weights combined for the severe initial health state. “Long” refers to the 5‐year durations

Figure 2 gives the mean age weights divided by three age groups (18–44, 45–60 and > 60) and for the full sample. The age groups are about equally large. These numbers show that the oldest group tends to give most weight to the 20‐year‐old group.

### 4.4 Reference‐dependence

We present the results from the reference‐dependence tests in Tables 6, 7, 8, 9. One can see that the division in the different types is often unbalanced, with Type 1 having a larger sample size than the other two Types for the mild condition. Similarly, Type 2 has a larger sample size for the severe condition. Therefore, we also performed an analysis on pooled datasets, where we combined the age weights of all conditions and classification systems. We did so for each starting level separately. The mean age weights for the mild condition are reported in Table 6 and the test results in Table 7. For the severe condition, these numbers are shown in Tables 8 and 9, respectively.

**TABLE 6:** Mean age weights (*w*_20_) mild initial health states, separated by gains and losses if RL = 1–2 for EQ‐5D, 1 for Health Utility Index (HUI3)

	Type 1	Type 2	Type 3
Mobility 3‐>1	0.63 (*n* = 114)	0.61 (*n* = 36)	0.45 (*n* = 9)
Hearing 2‐>1	0.67 (*n* = 115)	0.69 (*n* = 13)	0.52 (*n* = 37)
Anxiety 3‐>1	0.59 (*n* = 75)	0.67 (*n* = 64)	0.58 (*n* = 24)
Ambulation 2‐>1	0.66 (*n* = 119)	0.67 (*n* = 9)	0.63 (*n* = 32)
Mobility 3‐>1 (5 years)	0.63 (*n* = 112)	0.64 (*n* = 28)	0.41 (*n* = 18)
Hearing 2‐>1 (5 years)	0.70 (*n* = 112)	0.67 (*n* = 11)	0.51 (*n* = 39)
All combined	0.65 (*n* = 647)	0.65 (*n* = 161)	0.53 (*n* = 159)

**TABLE 7:** Results *t*‐tests mild initial health states (*p*‐values)

	Type 1 versus 2	Type 1 versus 3	Type 2 versus 3
Mobility 3‐>1	0.78	0.083	0.09
Hearing 2‐>1	0.71	<0.01	0.039
Anxiety 3‐>1	0.05 (opposite direction)	0.93	0.22
Ambulation 2‐>1	0.91	0.60	0.71
Mobility 3‐>1	0.80	<0.01	<0.01
Hearing 2‐>1	0.65	<0.01	0.11
All combined	0.79	<0.01	<0.01

**TABLE 8:** Mean age weights ($w_{20})$ severe initial health states, separated by gains and losses if RL = 1–3 for EQ‐5D and Health Utility Index (HUI3)

	Type 1	Type 2	Type 3
Mobility 5‐>3	0.51 (*n* = 32)	0.64 (*n* = 125)	0.55 (*n* = 2)
Hearing 4‐>2	0.52 (*n* = 67)	0.57 (*n* = 78)	0.57 (*n* = 14)
Anxiety 5‐>3	0.61 (*n* = 19)	0.63 (*n* = 134)	0.35 (*n* = 7)
Ambulation 4‐>2	0.67 (*n* = 43)	0.61 (*n* = 100)	0.64 (*n* = 14)
Mobility 5‐>3 (long)	0.57 (*n* = 35)	0.67 (*n* = 119)	0.41 (*n* = 8)
Hearing 4‐>2 (long)	0.67 (*n* = 57)	0.70 (*n* = 82)	0.45 (*n* = 22)
All combined	0.59 (*n* = 253)	0.64 (*n* = 638)	0.50 (*n* = 67)

**TABLE 9:** Results *t*‐tests severe initial health states (*p*‐values)

	Type 1 versus Type 2	Type 1 versus Type 3	Type 2 versus Type 3
Mobility 5‐>3	0.02	0.85	0.64
Hearing 4‐>2	0.24	0.52	0.98
Anxiety 5‐>3	0.75	0.03	<0.01
Ambulation 4‐>2	0.18	0.65	0.68
Mobility 5‐>3 (long)	0.06 (opposite dir.)	0.15	0.01
Hearing 4‐>2 (long)	0.41	<0.01	<0.01
All combined	0.02	0.02	<0.01

Table 6 makes clear that the weights for Type 1 and 2 are rather similar, whereas the weights for Type 3 are lower for all cases. However, due to a small number of respondents in several cells in Type 2 and 3, these differences are often nonsignificant. Table 7 indicates the differences between Type 3 and the other scenarios are especially significant for hearing and mobility, as well as the combined conditions, which has a considerably larger sample size. Types 1 and 2 do not differ significantly from each other, except of anxiety/depression, where the difference is in the opposite direction from what we expected (*p* = 0.05). Tables 8 and 9 give a similar picture, although now Type 3 differs from Types 1 and 2 for anxiety/depression in the predicted direction (*p* < 0.04).

Table 10 provides the mean values of *w*_20_ separated by the combination of Types for the two health conditions. For instance, the second column shows the mean values for the subset of respondents who regarded the treatment of the mild condition as a gain enlargement for the old and a loss reduction for the young (Type 1), while at the same time regarding the treatment of the severe condition to be a loss reduction for both age groups (Type 2). The results indicate an absence of the effect predicted by loss aversion (Type 1 vs. 2 and 3). Instead, the only significant difference is for the respondents who are trading off losses against gains for *both severities*, with the age weight being higher for mild health states than severe health states. This indicates that respondents who have a high RL for 20‐year‐olds and a low RL for 80‐year‐olds are giving more weight to the 20‐year‐olds when it concerns a mild initial state than when it concerns a severe state, despite that it is a loss reduction for the 20‐year‐olds in both tasks, and a gain enlargement in both tasks for the 80‐year‐olds. The same result was found (albeit only significant at the 10% level) for those respondents in Type 1 for the severe initial health and in Type 3 for the mild initial health.

**TABLE 10:** Mean age weights (*w*_20_) separated by type for both mild and severe health states and results of paired *t*‐tests for all versions combined^a^

	Type 1 for mild and Type 2 for severe		Type 1 for both mild and severe		Type 2 for both mild and severe		Type 3 for mild and Type 1 for severe		Type 3 for both mild and severe	
Severity	Mild	Severe	Mild	Severe	Mild	Severe	Mild	Severe	Mild	Severe
Attribute
Mobility	0.64 (*n* = 84)	0.67 (*n* = 83)	0.57 (*n* = 30)	0.53 (*n* = 29)	0.61 (*n* = 36)	0.61 (*n* = 37)	0.61 (*n* = 3)	0.26 (*n* = 3)	0.52 (*n* = 2)	0.55 (*n* = 2)
Hearing	0.66 (*n* = 57)	0.57 (*n* = 56)	0.67 (*n* = 58)	0.52 (*n* = 58)	0.69 (*n* = 13)	0.71 (*n* = 13)	0.64 (*n* = 9)	0.50 (*n* = 9)	0.39 (*n* = 14)	0.57 (*n* = 14)
Anxiety	0.58 (*n* = 60)	0.59 (*n* = 60)	0.60 *n* = 15)	0.59 (*n* = 15)	0.67 (*n* = 64)	0.64 (*n* = 64)	0.69 (*n* = 4)	0.68 (*n* = 4)	0.25 (*n* = 7)	0.35 (*n* = 7)
Ambulation	0.66 (*n* = 84)	0.63 (*n* = 84)	0.67 (*n* = 35)	0.69 (*n* = 35)	0.67 (*n* = 9)	0.49 (*n* = 9)	0.72 (*n* = 8)	0.60 (*n* = 8)	0.66 (*n* = 14)	0.64 (*n* = 14)
Mobility long dur.	0.64 (*n* = 82)	0.69 (*n* = 82)	0.60 (*n* = 30)	0.59 (*n* = 30)	0.64 (*n* = 28)	0.63 (*n* = 28)	0.43 (*n* = 5)	0.45 (*n* = 5)	0.43 (*n* = 8)	0.41 (*n* = 8)
Hearing long dur.	0.67 (*n* = 66)	0.71 (*n* = 66)	0.75 (*n* = 46)	0.71 (*n* = 46)	0.67 (*n* = 11)	0.68 (*n* = 11)	0.53 (*n* = 11)	0.51 (*n* = 11)	0.46 (*n* = 22)	0.45 (*n* = 22)
All combined	0.64 (*n* = 433)	0.65 (*n* = 431)	0.66 (*n* = 214)	0.60 (*n* = 213)	0.65 (*n* = 161)	0.63 (*n* = 162)	0.60 (*n* = 40)	0.52 (*n* = 40)	0.46 (*n* = 67)	0.50 (*n* = 67)
*p*‐value of paired *t*‐tests	*P* = 0.64		*P* < 0.01		*P* = 0.17		*P* = 0.06		*P* = 0.21	

^a^ Sample sizes for each cell in parentheses.

We also computed the average age of the respondents for each type. It turns out that this differs considerably between types, reflecting the positive correlation between respondent's age and perceived normal QoL level. That is, older respondents are more likely to consider both patient groups to be in the loss domain and hence are better represented in Type 2 (mean age 55.7 years for mild and 52.5 years for severe), whereas younger respondents are more inclined to consider both groups to be in the gain domain and therefore make up a larger part of Type 3 (mean age 43.5 years for mild and 43.7 years for severe). The mean age for Type 1 was in‐between with 51.9 years for mild and 51.6 years for severe.

Table 11 gives a decomposition of the choices in the first question (where the number of patients in each group was 100) for the different types for the mild initial health states (the results for the severe initial health states are comparable, except that a lower percentage preferred the young group in the first question, see Table B4 in Appendix B). The table also shows the mean age weights for the respondents preferring each group in this first question.

**TABLE 11:** Percentages choosing each age group when both are of equal size (100), including the mean age weights separated by respondents preferring the young and respondents preferring the old (mild initial health states)

3‐>1 (or 2‐>1 for HUI)	Prefer 100 young in Q1	Prefer 100 old in Q1	Total number of respon‐dents	Mean indifference of those preferring 20years	Mean w of those preferring 20years	Mean indifference of those preferring 80years	Mean w of those preferring 80years
Type 1	467 (72.1%)	181 (27.9%)	648	32 young = 100 old	0.76	100 young = 59 old	0.37
Type 2	126 (77.8%)	36 (22.2%)	162	35 young = 100 old	0.74	100 young = 52 old	0.34
Type 3	93 (58.5%)	66 (41.5%)	159	35 young = 100 old	0.74	100 young = 32 old	0.24

Abbreviation: HUI, Health Utilities Index.

A remarkable observation is that the average intensity of age weighting, that is, the amount of young (old) patients treated being regarded as equivalent to treat 100 old (young) patients, is higher among those who prefer the young than among those who prefer the old in the first two types, whereas this intensity is about the same in the third type. That is, the respondents preferring the 20‐year‐old group in the first question, on average value an accelerated treatment of about 35 young patients the same as accelerating the treatment of 100 old patients, whereas those respondents who prefer the 80‐year‐old group in the first question are indifferent between giving this treatment to 59 (Type 1) or 52 (Type 2) old patients and giving it to 100 young patients. If preference intensity was the same between those preferring the young and those preferring the old, one would expect the number of young patients for the former group to be about the same as the number of old patients for the latter group. This only happens for Type 3 respondents (i.e., 35 vs. 32).

## 5 DISCUSSION

This study found a substantial amount of age weighting, with QALY gains of young patient groups receiving about 1.7 times as much weight as QALY gains of old patient groups. These preferences for the young were neither affected by the nature of the health problems (i.e., mental or physical) nor by the duration of the health gains. We did find a positive correlation between the weight given to the young and the age of the respondents, which was significant for the severe condition. In addition, we have proposed an alternative account of age weighting, where reference points play a role in shaping preferences for the allocation of a fixed health care budget to one of two age groups. Outcomes falling below this reference point are seen as losses in this theory, and, due to loss aversion, receive more weight than outcomes above it, which are regarded as gains. However, the evidence was hardly supportive of loss aversion being a determinant of age weighting. Only the results for treating mild hearing problems corresponded to this explanation. Nevertheless, although not predicted by our theory, reference health levels turned out to be relevant for part of the patient age preferences. In particular, respondents with lower reference levels gave less priority to QALY improvements in 20‐year‐old patients relative to 80‐year‐old persons. That is, people who were trading off two gain enlargements (Type 3) showed less weighting toward the young than people who were trading off two loss reductions (Type 2), and less than people who were trading off loss reductions with gain enlargements (Type 1). A potential explanation for this finding might be that gains for older patients are considered less valuable than gains for younger patients, whereas losses are deemed problematic for both patient groups, causing less age weighting there. However, this explanation only holds for the comparison of Types 2 and 3; not for the comparison of Types 1 and 3. There it suggests that gain enlargements are more valuable than loss reductions for young patients, which would point toward gain seeking, that is, the opposite of loss aversion. In addition, the final health state reached, also in relation to for example, role functioning, may play a role here (Olsen, 2013). On the other hand, gain seeking would predict more age weighting in Type 2 than in Type 1, which we do not observe. Instead, people who are more optimistic about health might have a general tendency to care more about the health of the young relative to the old. This possibility would be an interesting future research venue.

Another interesting result is that, within the subset of the sample that considered the health improvements as gain enlargements for 80‐year‐old people and loss reductions for 20‐year‐olds, the age weight was significantly higher for the mild initial health state than for the severe initial health state. This suggests stronger preferences for the young for alleviations of mild health problems than for alleviations of severe health problems. Although on the face of it, loss aversion appears a plausible explanation for such a preference, our findings indicate that this is not the case, because according to their reference health levels, these respondents are comparing gain enlargements and loss reductions in both cases. Hence, alternative explanations need to be sought for this difference. One possibility is that special weight is given to treatments that regain full functioning for young persons; whereas, for older persons it is deemed less crucial to be without any problems. The fact that, in case of a severe initial health state, some problems will remain (even after treatment) may explain the lower amount of age weighting there. Take for example, the improvement from mobility from level 5 to level 3. Respondents might feel that level 3 is still too poor for 20‐year‐old persons and hence may prefer to give to 80‐year‐old persons for whom the improvement might provide more utility. Alternatively, respondents might consider the implemented initial states so severe that they feel both patient groups should be relieved from it with equal priority. Still, this reasoning does not explain the lack of a difference between these two tasks for the respondents in the other two scenarios (e.g., both tasks involving loss reductions for both the young and the old). Another interesting option to explore in future research is whether the reference point we used in our study is the most important reference point considered by the respondents. An alternative could for instance be acceptable (rather than normal) health (Brouwer et al., 2005; Péntek, Rojkovich, et al., 2014; Wouters et al., 2015; Zrubka et al., 2019).

If we decompose the sample into respondents giving more weight to the young than to the old and respondents with the opposite preferences, we find another interesting pattern. It appears that among respondents in Types 1 and 2, there is a large difference in the intensity of preference between those who prefer the young and those who give more weight to the old. In particular, the former group has a much higher preference intensity than the latter group. Instead, considering the respondents in Type 3, with low reference levels for both patient groups (i.e., respondents with a pessimistic view on normal QoL), we do not find such a distinction. Respondents who give more weight to the old than the young have a higher intensity of preference in this scenario. It therefore appears that for people who prefer the old, the intensity of these preferences is higher among those who are more pessimistic about normal QoL.

In a study on equity weights with regard to quality‐of‐life levels, Attema et al. (2015) found a substantial amount of loss aversion. Although we did not directly elicit loss aversion in the current study, our results did not support a reference‐dependent social welfare function as an explanation for age weighting. This stresses the importance of properly considering the context. Even though loss aversion has been found to be a robust phenomenon for both money and health, it may still be of less relevance in particular domains, such as trade‐offs between different cohorts. Other studies already found loss aversion to be of a lower magnitude for health than for money in the case of individual decisions (Attema et al., 2013, 2016, 2018; Rouyard et al., 2018).

Another result of our study that warrants more attention is the role of the age of the respondents. Older people clearly had a more positive idea of health at higher ages. This was also highlighted by the age composition of the respondents in the different scenarios, with the average respondent age of Type 2 (all loss reductions) considerably higher than of Type 1 (loss reduction for the young, gain enlargement for the old), which was again substantially higher than that of Type 3 (gain enlargement for both). Surprisingly, this partly translated into older respondents giving more weight to the young. Since almost all respondents were 20 years or older, they all have experience with their health at age 20, so that provides no reason for older respondents to rate normal QoL level of 20‐year‐olds higher. Two alternative explanations may be considered. First, if older people rate normal QoL at age 80 higher than younger people do, but still want to express a declining health profile over time, this may easily result in a relatively high indication of normal QoL at age 20. Second, as people grow old, they have obtained more experience with poorer health and, as a consequence (or with hindsight), then think that one's health at age 20 years is very good. However, since young people are usually less familiar with poorer health, even small health problems may be considered worse than what old people think (Lipman et al., 2019). Still, this finding is consistent with the more rapid deterioration of normal QoL with aging for older respondents than for younger respondents. That is, if someone regards it normal for elderly to be in poor health, they are more likely to give more weight to treat the young to get out of poor health than the old.

Turning to the comparison of mental and physical conditions, our findings are to some extent similar to those of Brouwer et al. (2005), Johri et al. (2005), and Richardson et al. (2017). Brouwer et al. (2005) reported that problems in the physical domains of the EQ‐5D system are considered to be more acceptable at older ages than the mental domain (anxiety/depression). Johri et al. (2005) observed age to be more important in treating infertility and lifesaving than depression. Richardson et al. (2017) reported that age weights decline faster with the age of recipients for physical conditions than for mental conditions. Although we corroborate their findings when it concerns normal QoL levels, it turns out these differences between mental and physical health problems do not translate into differences in age weighting, whereas according to loss aversion, age weighting would be predicted to be stronger for physical problems than mental problems. However, the apparent absence of loss aversion in our study is consistent with this lack of difference in age weighting between mental and physical age weighting.

One reason why we did not find robust evidence of loss aversion could be that our assumption that utilities are the same for gains and losses is not valid. Hence, future work might first elicit the utility functions for gains and losses separately and correct for them when estimating loss aversion. However, as the outcome of interest only considers five or six health levels, this is not straightforward. In addition, our design could only test if age weighting was different when comparing gains to losses than when comparing gains to gains or losses to losses. A confirmation of such a test would be supportive of the presence of loss aversion, but it cannot rule out other explanations, such as referenced‐dependent age weights instead of reference‐dependent utility. Because our design could not distinguish between these two explanations, future research is encouraged to elicit the utility function and loss aversion index alongside age weights, in order to test to what extent reference‐dependent age weighting and reference‐dependent utility explain age‐dependent priority setting.

Another limitation of our study is that our questions to measure age weighting were not tailor‐made to the respondents' individual reference levels. Follow‐up research could adopt such an approach to test for sign‐dependence with more power. Alternatively, experimenters could try to control the different reference points by using exogenous instead of endogenous reference points. This would avoid the potential for collinearity between age weighting preferences and the respondents' type (as determined by their reference levels), which may have distorted our study. However, a drawback of using induced reference points is that one cannot be certain that the respondents indeed apply these reference points or keep their own reference points in mind.

A further shortcoming of this study is that the number of respondents assigned to each specific health condition was limited, leading to a lack of power in testing for differences between some types. Given the novelty of our approach, we did not have sufficient information to conduct a proper sample size calculation. Therefore, this study should be regarded as a first pilot test of the presence of reference‐dependence utility in age weighting. Future research is needed to provide more robust evidence about this hypothesis. Statistical power can be increased by focusing on one or two health problems, and increasing the sample size, making use of the distribution of reference levels reported in this paper. Moreover, it could use personal interviews as a mode of administration instead of our use of an online survey, which will likely increase effort, improve task comprehension and reduce noise.

We conclude that age weighting is a robust phenomenon in deciding about allocation of quality‐of‐life improvements, but its magnitude is smaller for people with lower reference levels. It is not affected by an improvement being a gain enlargement for older patients while being a loss reduction for younger patients. Hence, people who are pessimistic about what level of health can be viewed as normal, have weaker preferences for the young than those who are more optimistic. This suggests reference points are still an important determinant of age‐related equity weighting.

## CONFLICT OF INTEREST

The authors declare to have no conflicts of interest.

## Supporting information

Supporting Information S1

## Data Availability

The data that support the findings of this study are available from the corresponding author upon reasonable request.

## ENDNOTES

^1^
Note that there is a potential collinearity issue here, because the age preferences of the respondent may be related to their type. We return to this issue in the Discussion.

^2^
We make the usual assumption that *U*(*q*) is a cardinal function, which could be anchored such that *U*(death) = 0 and *U*(perfect health) = 1, as commonly done. If this function is continuous, the reference‐dependent model can be applied to it the same way as for monetary outcomes.

^3^
However, we admit that 5 min is an arbitrary threshold for speeding and does not automatically imply low‐quality responses.

^4^
It could be argued that the results for the reduced sample should be reported in the main text instead of an appendix in order to reduce noise. However, since there are no important differences between those respondents who failed the dominance test and those who passed it, we feel we do not have good reasons to exclude this part of the sample.

## APPENDIX A EXPERIMENTAL INSTRUCTIONS AND SELECTED QUESTIONS

### General instructions

Dear respondents,

Thank you for your cooperation in this research. The study has to do with the distribution of health in The Netherlands. First, we ask you some general questions, after which several questions with regard to this research will be asked. These questions are equipped with an explanation. The survey takes no longer than half an hour.

### Questions on socio‐demographics

1. What is your gender?
2. What is your age?
3. What is your highest completed education?
4. Consider the beneath thermometer. 100 indicates perfect health and 0 is a health state as poor as death. How do you value your own health today on this scale?

### Elicitation of perceived normal QoL states

Consider the beneath table. This table shows a number of possible hearing conditions.

**Table:**

Level	Description
1	Able to hear what is said in a group conversation with at least three other people, without a hearing aid.
2	Able to hear what is said in a conversation with one other person in a quiet room without a hearing aid, but requires a hearing aid to hear what is said in a group conversation with at least three other people.
3	Able to hear what is said in a conversation with one other person in a quiet room with a hearing aid, and able to hear what is said in a group conversation with at least three other people, with a hearing aid.
4	Able to hear what is said in a conversation with one other person in a quiet room, without a hearing aid, but unable to hear what is said in a group conversation with at least three other people even with a hearing aid.
5	Able to hear what is said in a conversation with one other person in a quiet room with a hearing aid, but unable to hear what is said in a group conversation with at least three other people even with a hearing aid.
6	Unable to hear at all.

- Which of those situations do you think is normal at 80?
- Which of those situations do you think is normal at 20?

### Instructions PTO task

- The following questions each time describe two different treatments (A and B).
- Suppose you are a policy maker and have to decide on behalf of Dutch society.
- Both treatments are equally expensive, but a limited amount of money is available to spend on one of two treatments.
- You don't belong to the patient group yourself but you have to make choices on behalf of society.

The two treatments are shown in a table. The health change through this treatment is indicated on top of the table and is underlined. The size of the group and the age of the group members are in the table. A change in the size of the group size or age of the group compared to the previous question is **printed in bold**.

The following two questions are practice questions and after that the research questions will follow.

**Practice question 1**.

- Consider one group of 100 persons of age 25 years and another group of 100 persons of age 25 years.
- Except for their age, the persons in these groups do not differ in any other way,
- Assume both groups have hearing problems (level 5 in the table).
- The problems disappear in both groups by means of a standard treatment after 6 months (Treatment A).
- A new, better, treatment is also available, which causes the illness to disappear 3 months sooner (Treatment B).
- However, that better treatment cannot be given to everyone.
- You can give Treatment B to:
  ‐ 60 persons of 25 years old (Group 1)
  ‐ 100 persons of 25 years old (Group 2)

- To which group would you give this new treatment? (The other group then automatically gets Treatment A).

Health change because of treatment: from Level 5 to Level 1.

**Table:**

	Group 1	Group 2
Number of persons	60	100
Age of the group members	25 years	25 years

*Feedback given after choice for dominated option (i.e., Group 1)*

You have chosen for Group 1: a group of 60 persons of 25 years old gets Treatment B, causing their hearing to improve 3 months sooner. The group of 100 persons of 25 years old gets Treatment A, causing their hearing to improve after only 6 months. If you opt for Group 1, you are thus only treating the smallest group of 60 25‐year‐old people with Treatment B, instead of the large group of hundred 25‐year‐old people. Do you really prefer to help fewer instead of more people of the same age, with the same health, and with the same other characteristics?

*Example of a choice question*.

*Example of a question to determine the indifference point*.

*Overview of other health state classifications*

**Table:**

Level	Anxiety/depression
1	Not anxious or depressed.
2	Slightly anxious or depressed.
3	Moderately anxious or depressed.
4	Severely anxious or depressed.
5	Extremely anxious or depressed.

Level	Mobility
1	No problems walking about
2	Some problems walking about
3	Moderate problems walking about
4	Severe problems walking about
5	Not able to walk about

Level	Ambulation
1	Able to walk around the neighborhood without difficulty, and without walking equipment.
2	Able to walk around the neighborhood with difficulty; but does not require walking equipment or the help of another person.
3	Able to walk around the neighborhood with walking equipment, but without the help of another person.
4	Able to walk only short distances with walking equipment, and requires a wheelchair to get around the neighborhood.
5	Unable to walk alone, even with walking equipment. Able to walk short distances with the help of another person, and requires a wheelchair to get around the neighborhood.
6	Cannot walk at all.

## APPENDIX B SUPPLEMENTARY ANALYSES

**TABLE B1:** Median age weights of 20‐year‐olds (*w*_20_, interquartile ranges in parentheses)

Delay of health improvement				
Task	3 months or 6 months		3 months or 5 years	
	3→1 (EQ5d)	5→3 (EQ5d)	3→1 (EQ5d)	5→3 (EQ5d)
	2→1 (HUI3)	4→2 (HUI3)	2→1 (HUI3)	4→2 (HUI3)
EQ‐5D—mobility	0.67 (0.43–0.89)	0.65 (0.42–0.89)	0.67 (0.41–0.89)	0.69 (0.47–0.89)
EQ‐5D—anxiety	0.67 (0.44–0.89)	0.67 (0.50–0.83)		
HUI—ambulation	0.69 (0.50–0.89)	0.67 (0.49–0.89)		
HUI—hearing	0.67 (0.50–0.89)	0.53 (0.32–0.83)	0.68 (0.49–0.89)	0.72 (0.50–0.89)

Abbreviation: HUI, Health Utilities Index.

### Results of analyses for alternative classifications

**TABLE B2:** Mean age weights mild initial health states, separated by gains and losses if RL = 1 for both EQ‐5D and Health Utility Index (HUI3)

*w*_20_			
	Gains versus losses Type 1	Losses versus losses Type 2	Gains versus gains Type 3
Mobility 3‐>1	0.62 (*n* = 137)	0.42 (*N* = 3)	0.59 (*n* = 20)
Hearing 2‐>1	0.67 (*n* = 115)	0.69 (*N* = 13)	0.52 (*n* = 37)
Anxiety 3‐>1	0.60 (*n* = 84)	0.71 (*N* = 19)	0.62 (*n* = 59)
Ambulation 2‐>1	0.66 (*n* = 119)	0.67 (*N* = 9)	0.63 (*n* = 32)
Mobility 3‐>1	0.63 (*n* = 127)	0.59 (*N* = 7)	0.49 (*n* = 27)
Hearing 2‐>1	0.70 (*n* = 112)	0.67 (*N* = 11)	0.51 (*n* = 39)
All combined	0.65 (*n* = 694)	0.67 (*n* = 62)	0.57 (*n* = 214)

**TABLE B3:** Mean age weights severe initial health states, separated by gains and losses if RL = 1–4 for EQ‐5D and for Health Utility Index (HUI3)

*w*_20_			
	Type 1	Type 2	Type 3
Mobility 5‐>3	0.11 (*n* = 4)	0.62 (*n* = 157)	‐ (*n* = 0)
Hearing 4‐>2	0.54 (*n* = 34)	0.55 (*n* = 121)	0.40 (*n* = 5)
Anxiety 5‐>3	0.55 (*n* = 5)	0.63 (*n* = 156)	0.12 (*n* = 2)
Ambulation 4‐>2	0.60 (*n* = 10)	0.63 (*n* = 144)	0.73 (*n* = 3)
Mobility 5‐>3	0.46 (*n* = 9)	0.65 (*n* = 151)	0.31 (*n* = 2)
Hearing 4‐>2	0.60 (*n* = 24)	0.68 (*n* = 130)	0.43 (*n* = 6)
All combined	0.53 (*n* = 86)	0.63 (*n* = 859)	0.42 (*n* = 18)

**TABLE B4:** Percentages choosing each age group when both are of equal size (100), including the mean age weights separated by respondents preferring the young and respondents preferring the old (severe initial health states)

5 ‐> 3 (or 4‐>2 for HUI)	Prefer 100 young in Q1	Prefer 100 old in Q1	Total number of respon‐dents	Mean indifference of those preferring 20years	Mean w of those preferring 20years	Mean indifference of those preferring 80years	Mean w of those preferring 80years
Type 1	162 (63.8%)	92 (36.2%)	254	35 young = 100 old	0.74	100 young = 47 old	0.32
Type 2	450 (70.3%)	190 (29.7%)	640	33 young = 100 old	0.75	100 young = 59 old	0.37
Type 3	35 (52.2%)	32 (47.8%)	67	41 young = 100 old	0.71	100 young = 39 old	0.28

Abbreviation: HUI, Health Utilities Index.
