# Supplementary material for: Reference‐dependent age weighting of quality‐adjusted life years
Source: Health Econ. 2022 Sep 4;31(12):2515–36. doi: 10.1002/hec.4593 (PMC9826257; doi:10.1002/hec.4593)
Supplement: Supplementary file 1 — Supporting Information S1 [file HEC-31-2515-s001.docx]

**Web Appendix. Results of robustness analyses.**

The beneath tables reproduce the tables of the manuscript (with the corresponding number) when the respondents completing the survey in less than 5 minutes and/or violating dominance in the practice question and not willing to revise their choice are excluded from the analysis.

*Table W4. Average perceived normal levels per attribute for 20- and 80-year-olds (standard deviations in parentheses).*

| Age of patients | Mobility  (scale 1-5)  A+E | Anxiety/Depression  (scale 1-5)  C | Hearing  (scale 1-6)  B+F | Ambulation  (scale 1-6)  D |
| --- | --- | --- | --- | --- |
| 20 years | 1.30 (0.80) | 1.56 (0.93) | 1.32 (0.94) | 1.34 (1.01) |
| 80 years | 3.03 (0.83) | 2.59 (0.98) | 3.32 (1.25) | 2.98 (1.00) |

*Table W5. Mean estimates of* $w_{20}$ *(standard deviations in parentheses)*

**Delay of health improvement**

|  | **3 months or 6 months** | | **3 months or 5 years** | |
| --- | --- | --- | --- | --- |
| **Health improvement**  **Task** | 3🡪1 (EQ5D)  2🡪1 (HUI3) | 5🡪3 (EQ5D)  4🡪2 (HUI3) | 3🡪1 (EQ5D)  2🡪1 (HUI3) | 5🡪3 (EQ5D)  4🡪2 (HUI3) |
| EQ-5D – mobility | 0.60 (0.28)  N=144 | 0.61 (0.28)  N=143 | 0.62 (0.27)  N=146 | 0.64 (0.27)  N=146 |
| EQ-5D – anxiety | 0.63 (0.26)  N=143 | 0.63 (0.24)  N=143 |  |  |
| HUI – ambulation | 0.64 (0.26)  N=125 | 0.62 (0.27)  N=125 |  |  |
| HUI – hearing | 0.65 (0.24)  N=129 | 0.55 (0.28)  N=129 | 0.66 (0.25)  N=130 | 0.67 (0.26)  N=130 |

*Table W6. Mean age weights* ${(w}_{20})$ *mild initial health states, separated by gains and losses if RL=1-2 for EQ-5D, 1 for HUI3*

|  | Type 1 | Type 2 | Type 3 |
| --- | --- | --- | --- |
| Mobility 3->1 | 0.62 (n=102) | 0.61 (n=28) | 0.49 (n=8) |
| Hearing 2->1 | 0.67 (n=102) | 0.67 (n=9) | 0.47 (n=16) |
| Anxiety 3->1 | 0.61 (n=66) | 0.66 (n=58) | 0.62 (n=16) |
| Ambulation 2->1 | 0.64 (n=103) | 0.65 (n=7) | 0.62 (n=15) |
| Mobility 3->1 | 0.64 (n=102) | 0.65 (n=26) | 0.43 (n=15) |
| Hearing 2->1 | 0.69 (n=102) | 0.65 (n=10) | 0.47 (n=17) |
| All combined | 0.65 (n=577) | 0.65 (n=138) | 0.52 (n=87) |

*Table W7. Results t-tests mild initial health states (p-values)*

|  | Type 1 vs. 2 | Type 1 vs. 3 | Type 2 vs. 3 |
| --- | --- | --- | --- |
| Mobility 3->1 | 0.92 | 0.23 | 0.25 |
| Hearing 2->1 | 0.99 | <0.01 | 0.04 |
| Anxiety 3->1 | 0.20 | 0.87 | 0.55 |
| Ambulation 2->1 | 0.92 |  | 0.78 |
| Mobility 3->1 | 0.77 | 0.71 | 0.014 |
| Hearing 2->1 | 0.62 | <0.01 | 0.13 |
| All combined | 0.92 | <0.01 | <0.01 |

*Table W8. Mean age weights (*$w_{20})$ *severe initial health states, separated by gains and losses if RL=1-3 for EQ-5D and HUI3*

|  | Type 1 | Type 2 | Type 3 |
| --- | --- | --- | --- |
| Mobility 5->3 | 0.50 (n=30) | 0.64 (n=106) | 0.55 (n=2) |
| Hearing 4->2 | 0.52 (n=53) | 0.57 (n=68) | 0.60 (n=7) |
| Anxiety 5->3 | 0.59 (n=15) | 0.64 (n=120) | 0.44 (n=4) |
| Ambulation 4->2 | 0.66 (n=31) | 0.60 (n=85) | 0.63 (n=7) |
| Mobility 5->3 | 0.59 (n=29) | 0.67 (n=110) | 0.46 (n=7) |
| Hearing 4->2 | 0.67 (n=50) | 0.69 (n=71) | 0.43 (n=9) |
| All combined | 0.60 (n=180) | 0.63 (n=594) | 0.53 (n=37) |

*Table W9. Results t-tests severe initial health states (p-values)*

|  | Type 1  vs. Type 2 | Type 1  vs. Type 3 | Type 2  vs. Type 3 |
| --- | --- | --- | --- |
| Mobility 5->3 | 0.02 | 0.53 | 0.64 |
| Hearing 4->2 | 0.33 | 0.54 | 0.80 |
| Anxiety 5->3 | 0.45 | 0.32 | 0.09 |
| Ambulation 4->2 | 0.33 | 0.82 | 0.79 |
| Mobility 5->3 | 0.13 | 0.18 | 0.04 |
| Hearing 4->2 | 0.56 | 0.02 | <0.01 |
| All combined | 0.025 | 0.16 | <0.01 |

*Table W10. Mean age weights* ${(w}_{20})$ *separated by type for both mild and severe health states and results of paired t-tests for all versions combined**

|  | Type 1 for mild  Type 2 for severe | | Type 1 for both mild and severe | | Type 2 for both mild and severe | | Type 3 for mild and Type 1 for severe | | Type 3 for both mild and severe | |
| --- | --- | --- | --- | --- | --- | --- | --- | --- | --- | --- |
| Severity  Attribute | Mild | Severe | Mild | Severe | Mild | Severe | Mild | Severe | Mild | Severe |
| Mobility | 0.64 (n=74) | 0.66  (n=73) | 0.57 (n=28) | 0.53  (n=27) | 0.61 (n=28) | 0.62 (n=29) | 0.61 (n=3) | 0.26 (n=3) | 0.52 (n=2) | 0.55 (n=2) |
| Hearing | 0.66 (n=53) | 0.58  (n=53) | 0.68  (n=49) | 0.53 (n=49) | 0.67 (n=9) | 0.69 (n=9) | 0.59 (n=4) | 0.48 (n=9) | 0.36 (n=7) | 0.60 (n=7) |
| Anxiety | 0.61 (n=53) | 0.61  (n=53) | 0.57 (n=13) | 0.58 (n=13) | 0.66 (n=58) | 0.65 (n=58) | 0.67 (n=2) | 0.67 (n=2) | 0.31 (n=4) | 0.44 (n=4) |
| Ambulation | 0.64 (n=76) | 0.61  (n=76) | 0.67 (n=27) | 0.68 (n=27) | 0.65 (n=7) | 0.57 (n=7) | 0.66 (n=4) | 0.47 (n=4) | 0.67 (n=7) | 0.63 (n=7) |
| Mobility long dur. | 0.64 (n=77) | 0.69  (n=77) | 0.63 (n=25) | 0.61  (n=25) | 0.65 (n=26) | 0.64  (n=26) | 0.35 (n=4) | 0.42 (n=4) | 0.48 (n=7) | 0.46 (n=7) |
| Hearing long dur. | 0.65 (n=60) | 0.69  (n=60) | 0.75  (n=42) | 0.71 (n=42) | 0.65 (n=10) | 0.68 (n=10) | 0.51 (n=8) | 0.45 (n=8) | 0.44 (n=9) | 0.43 (n=9) |
| All combined | 0.64 (n=393) | 0.64 (n=392) | 0.66 (n=184) | 0.61 (n=183) | 0.65 (n=138) | 0.64 (n=139) | 0.55 (n=25) | 0.45 (n=25) | 0.47 (n=36) | 0.52 (n=36) |
| p-value of paired t-tests | P=0.74 | | P<0.01 | | P=0.57 | | P=0.10 | | P=0.28 | |

*Sample sizes for each cell in parentheses.

*Table W11. Percentages choosing each age group when both are of equal size (100), including the mean age weights separated by subjects preferring the young and subjects preferring the old (mild initial health states)*

| 3->1  (or 2->1 for HUI) | Prefer 100 young in Q1 | Prefer 100 old in Q1 | Total number of respon-dents | Mean indifference of those preferring 20y | Mean w of those preferring 20y | Mean indifference of those preferring 80y | Mean w of those preferring 80y |
| --- | --- | --- | --- | --- | --- | --- | --- |
| Type 1 | 418 (72.3%) | 160 (27.7%) | 578 | 32y = 100o | 0.76 | 100y = 59o | 0.37 |
| Type 2 | 107 (77.0%) | 32 (23.0%) | 139 | 35y = 100o | 0.74 | 100y = 54o | 0.35 |
| Type 3 | 50 (57.5%) | 37 (42.5%) | 87 | 37y=100o | 0.73 | 100y = 30o | 0.23 |

*Table WB4. Percentages choosing each age group when both are of equal size (100), including the mean age weights separated by subjects preferring the young and subjects preferring the old (severe initial health states)*

| 5->3  (or 4->2 for HUI) | Prefer 100 young in Q1 | Prefer 100 old in Q1 | Total number of respon-dents | Mean indifference of those preferring 20y | Mean w of those preferring 20y | Mean indifference of those preferring 80y | Mean w of those preferring 80y |
| --- | --- | --- | --- | --- | --- | --- | --- |
| Type 1 | 131 (62.7%) | 78 (37.3%) | 209 | 33y = 100o | 0.75 | 100y = 47o | 0.32 |
| Type 2 | 395 (70.3%) | 167 (29.7%) | 562 | 33y = 100o | 0.75 | 100y = 61o | 0.38 |
| Type 3 | 19 (52.8%) | 17 (47.2%) | 36 | 41y = 100o | 0.71 | 100y = 43o | 0.30 |
